# Intra-aortic balloon pump in patients with cardiogenic shock complicating myocardial infarction: a systematic review and meta-analysis of randomized trials (protocol)

**DOI:** 10.1186/2046-4053-3-24

**Published:** 2014-03-12

**Authors:** Sultan Altayyar, Bram Rochwerg, Sami Alnasser, Awad Al-omari, Bandar Baw, Alison Fox-Robichaud, Waleed Alhazzani

**Affiliations:** 1 Department of Medicine, Division of Critical Care, McMaster University, Hamilton, Canada; 2 Department of Critical Care Medicine, Prince Sultan Military Medical City, Riyadh, Saudi Arabia; 3 Department of Cardiology, St Michael Hospital, University of Toronto, Toronto, Canada; 4 Department of Critical Care, Security Forces Hospital, Riyadh, Saudi Arabia; 5 Department of Emergency Medicine, McMaster University, Hamilton, Canada

**Keywords:** cardiogenic shock, intra-aortic balloon pump, meta-analysis, myocardial infarction, systematic review

## Abstract

**Background:**

Cardiogenic shock is the leading cause of death in patients with acute myocardial infarction. Despite significant advancements in health technology and research, hospital mortality approaches 50%. The intra-aortic balloon pump is a mechanical hemodynamic assist device that has been used for over 40 years in the management of patients with cardiogenic shock. A recent randomized trial suggests that the use of intra-aortic balloon pumps does not reduce mortality in patients with ischemic cardiogenic shock.

**Methods:**

We plan to search MEDLINE, EMBASE, and the Cochrane Trial Registry for potentially eligible randomized trials that compare the use of intra-aortic balloon pump with no mechanical device support in patients with cardiogenic shock. No date, language or journal limitations will be applied. Two reviewers will independently screen and identify eligible studies using predefined eligibility criteria. Data abstraction will be done independently and in duplicate. We plan to use RevMan software to generate pooled estimates across included studies, using the previously published method of DerSimonian and Laird. We will report pooled estimates as risk ratios with 95% confidence intervals for binary outcomes, and as mean differences with 95% confidence intervals for continuous outcomes. We will assess the quality of evidence using the Grading of Recommendations Assessment, Development and Evaluation (GRADE) approach.

**Discussion:**

The aim of this systematic review and meta-analysis is to summarize the available evidence on the efficacy of the intra-aortic balloon pump in cardiogenic shock.

Systematic review registration: PROSPERO CRD42014007056.

## Background

### Description of the condition

Cardiogenic shock occurs as a consequence of cardiac pump failure. Pump failure can lead to decreased cardiac output and a subsequent increase in systemic vascular resistance, in order to maintain perfusion of vital organs. Cardiogenic shock is defined as persistent hypotension (systolic blood pressure <80 to 90 mmHg or mean arterial pressure 30 mmHg lower than baseline) with severe reduction in the cardiac index (<1.8 l.min^−1^.m^2^ without support or <2.0 to 2.2 l.min^−1^.m^2^ with support) and adequate or elevated ventricular filling pressures. Cardiogenic shock complicates approximately 5% of myocardial infarctions. Despite utilization of an early revascularization strategy and advancing health care, cardiogenic shock remains the leading cause of death in this population with a hospital mortality rate approaching 40 to 50% [1,2]. Intra-aortic balloon pump (IABP) counterpulsation is a commonly used mechanical hemodynamic assist method. However, its role in cardiogenic shock has been the subject of ongoing debate. Improved hemodynamics using an IABP in patients with cardiogenic shock have been reported anecdotally by care providers, with registry-based observational studies [3,4] suggesting potential benefit. However, a recent randomized trial showed no effect on 30-day mortality using an IABP compared with standard of care.

### Description of the intervention

IABP represents one type of mechanical hemodynamic support device, and it has emerged as the single most widely used circulatory assist device worldwide. The effect of IABP on coronary blood flow is variable. Some studies found little or no change in coronary blood flow [5-7] while others noted a significant increase [8,9].

### How the intervention might work

When the balloon inflates during diastole, blood is displaced into the proximal aorta. Subsequently, rapid balloon deflation during systole reduces aortic volume (afterload) by creating a vacuum-like effect. These effects are variable, and may depend on the volume of the balloon, position in the aorta, heart rate, rhythm, and other factors [8].

The hemodynamic effects of IABP may include a reduction in systolic blood pressure and an increase in aortic diastolic pressure, resulting in higher coronary blood flow. The net result is a reduced heart rate and pulmonary capillary wedge pressure and an increased cardiac output [8].

### Why this review is important

Since the publication of the most recent systematic review on this topic [10], a large randomized controlled trial (RCT) has been published [2]. Although the results of this RCT do not support the use of IABP in patients with cardiogenic shock, an updated review is needed, to summarize and assess the quality of available evidence.

### Objectives

We plan to conduct a systematic review and meta-analysis of RCTs to investigate the potential benefits and risks of using IABP in patients with cardiogenic shock secondary to acute myocardial infarction.

## Methods

### Types of study

We will include parallel group RCTs with no methodological quality restriction. Quasirandomized (pseudorandomized) trials and crossover studies will be excluded.

### Types of participant

The population of interest includes adult patients (age ≥18 years old) with cardiogenic shock (excluding mechanical cardiac complications) complicating acute myocardial infarction. Studies should be conducted in either intensive care unit or coronary care unit settings.

### Types of intervention

The intervention of interest is IABP counterpulsation. Studies that used other cardiac support devices, such as a left ventricular assist device or an extracorporeal membrane oxygenator are not eligible. The control group should be standard medical therapy only; studies comparing IABP with other support devices will be excluded.

### Types of outcome measure

#### Primary outcomes

Our primary outcome measure is all-cause mortality at hospital discharge. If this is not available, the longest reported mortality will be used (for example, if both 30- and 90-day mortality are reported, we will use data on 90-day mortality in the analysis of the primary outcome).

#### Secondary outcomes

Secondary outcome measures include:

1. Length of stay (days) in intensive or coronary care unit;

2. Stroke (ischemic or hemorrhagic);

3. Re-infarction;

4. Limb ischemia;

5. Clinically significant bleeding (defined as any bleeding that requires transfusion of more than two units of blood, or that is associated with hemodynamic instability not explained by other conditions).

### Search methods for identification of studies

#### Electronic searches

We will search the electronic databases MEDLINE and EMBASE and the Cochrane Trial Registry for eligible articles from inception to November 2013. We plan to use search terms that include IABP, cardiogenic shock, and a sensitive RCT filter for each database (Additional file 1).

#### Searching other resources

Two reviewers will independently search reference lists of review articles and systematic reviews for eligible articles. Abstracts in conferences and proceedings will be searched using a database provided through McMaster University’s electronic library, PapersFirst [11].

### Data collection and analysis

After identification of potentially relevant articles, two reviewers (SA) and (AA) will independently screen all citations and references using specific eligibility criteria. The kappa statistic will be used to measure agreement between reviewers [12]. Disagreement will be resolved by discussion and consensus, with the help of a third reviewer (WA) when required.

### Selection of studies

We will apply the following eligibility criteria:

1. Population: adult (18 years or older) patients with cardiogenic shock secondary to acute myocardial infarction;

2. Intervention: IABP along with standard medical therapy compared with standard medical therapy alone;

3. Outcomes (at least one): all-cause mortality, ICU length of stay, stroke, limb ischemia, or clinically significant bleeding;

4. Design: RCT. Quasi-randomized (pseudo-randomized) and crossover studies will be excluded.

### Data extraction and management

Data extraction will be done independently and in duplicate using predesigned data abstraction forms (Additional file 2). Abstracted data will contain: study title, authors’ information, relevant demographic data, the type of intervention and control, definitions and numerical data for outcomes of interest and data on methodologic quality for each included study. Disagreement will be resolved by discussion and consensus with help of a third reviewer (WA) when required.

### Assessment of risk of bias in included studies

In duplicate and independently, two reviewers will assess the methodological quality of individual trials utilizing the Cochrane tool for assessing risk of bias [13].

For each included study, we will provide a description, comment, and judgment of ‘Yes’, ‘Unclear’, or ‘No’ for each of the following items:

#### Adequate sequence generation (selection bias)

Sequence generation will be considered adequate if it was generated by computer or using published tables of random numbers. Coin-tossing, dice-throwing, and dealing shuffled cards will also be considered adequate methods of sequence generation.

#### Allocation sequence concealment (selection bias)

The concealment of the allocation sequence will be considered adequate when specific methods have been implemented to undoubtedly protect knowledge of the allocation before and until the participant was assigned to one of the trial arms.

#### Blinding of participants and researchers (performance bias)

Owing to the nature of the intervention, it will be impossible to blind patients, physicians, and caregivers. Risk of bias due to blinding will be assessed for each of the outcomes within each included study. For objective outcomes, such as mortality, stroke, and limb ischemia, the effect of lack of blinding is unlikely to bias the results.

#### Blinding of outcome assessment (detection bias)

Blinding will be considered adequate if outcome assessors and adjudicators are all blinded. Risk of bias due to blinding will be assessed for each of the outcomes within each included study.

#### Incomplete outcome data assessed (attrition bias)

We will assess studies for attrition bias whenever data is available on the number of patients at different stages of the study.

#### Free of selective reporting (reporting bias)

Selective outcome reporting will be assessed whenever the protocol of the study is available (as a separate publication of the protocol, as a registered protocol in electronic clinical trials registers, or as method section in a preliminary or abstract publication of the same study).

### Free other bias

Other sources of potential bias will be considered (for example, stopping trials early for benefit, baseline imbalance, or blocked randomization in unblended trials) The judgment for each category will be made, taking into consideration the effect of this domain across outcomes for a single trial. The following categories will be considered: low risk of bias: when bias is not present or, if present, is unlikely to alter the results seriously; unclear risk of bias: when the risk of bias raises some doubt about the results or the information reported does not allow for proper assessment; and high risk of bias: when bias may seriously alter the results and interpretation.

The overall risk of bias for an individual study will be categorized as ‘low’ (if the risk of bias is low in all domains), ‘unclear’ (if the risk of bias is unclear in at least one domain, with no high risk of bias domains), or ‘high’ (if the risk of bias is high in at least one domain). Agreement will be reached by consensus or by consulting a third reviewer (AFR).

### Measures of treatment effect

We will use RevMan 5.2 to conduct the meta-analyses. We plan to report pooled outcomes as risk ratios with 95% confidence intervals (CIs) for binary outcomes, and as mean differences with 95% CIs for continuous outcomes. Applying inverse variance weighting and the methods of DerSimonian and Laird [14], a random-effects model will be used, except if we include three or RCTs or fewer, or if a dominant trial is included (weight >50%). In the case of low event rates (less than 1%), the Peto odds ratio will be used to pool binary outcomes [15]. The number needed to treat will be derived from the pooled risk ratios, using the approach recommended by the Cochrane collaboration. When data are not suitable for pooling we will describe them qualitatively.

### Dealing with missing data

We will contact the authors of the primary studies for additional information on missing data. If this approach is not successful, we will analyze only the available data and discuss the potential impact of missing data on the findings of the review in the discussion section.

### Assessment of heterogeneity

We will assess for heterogeneity between studies using the Mantel-Haenszel χ^2^ statistic (*P* < 0.01 indicating substantial heterogeneity) and the *I*^2^ statistic [16]. We consider *I*^2^ > 50% to indicated a significant heterogeneity worthy of investigation. In the case of a significant statistic (for example, *I*^2^ > 80%) or clinical heterogeneity that is not explained by subgroup or sensitivity analysis we will not conduct a meta-analysis, and will instead describe the data qualitatively.

### Assessment of reporting biases

We will assess publication bias visually using a funnel plot generated using RevMan 5.1 software, and statistically using the Egger test [17]. If fewer than ten RCTs are included, we cannot reliably assess for publication bias.

### Subgroup analysis and investigation of heterogeneity

To explore significant heterogeneity, when possible we will conduct the following subgroup analyses:

1. Risk of bias: high versus low risk of bias, hypothesizing that high risk of bias studies will have a larger effect size;

2. Age: younger than 50 years old versus 50 years and older, hypothesizing that the younger age group will have a larger treatment effect;

3. Use of fibrinolysis versus percutaneous coronary intervention (PCI), hypothesizing that the use of PCI will attenuate the effect of intervention;

4. Infarct-related artery or territory (anterior versus others);

5. History of pre-existing myocardial infarction versus no history of myocardial infarction;

6. Timing of IABP insertion: IABP insertion before PCI versus after PCI.

We anticipate that all subgroup analyses will be challenging, owing to the lack of data and the anticipated small number of included studies.

### Sensitivity analysis

For the primary outcome (mortality), when possible we will conduct the following sensitivity analyses:

1. Random-effects, or fixed effect model;

2. Excluding studies published as abstracts;

3. Excluding high risk of bias studies.

We will describe the effects (if any) of sensitivity analyses on the overall results in the results section.

### Assessing the quality of evidence

We will use the Grading of Recommendations Assessment, Development and Evaluation (GRADE) approach to assess the quality of evidence for each outcome [18] and to assess the overall quality of evidence. We will judge the quality of evidence based on five criteria: risk of bias, inconsistency, imprecision, indirectness, and publication bias. Based on these criteria, the quality of evidence judgment could range from very low to high.

## Discussion

This systematic review will identify and synthesize evidence examining the potential benefits and harms of using IABP in patients with cardiogenic shock. Given the emergence of new evidence, and the lack of an updated systematic review, this review will help in summarizing the available evidence, both quantitatively and qualitatively.

## Abbreviations

CI: confidence interval; GRADE: grading of recommendations assessment, development and evaluation; IABP: intra-aortic balloon pump; PCI: percutaneous coronary intervention; RCT: randomized controlled trial.

## Competing interests

The authors declare that they have no competing interests.

## Authors’ contributions

WA and SA conceived the idea and designed search strategy and data abstraction forms. WA, SA, BR, SA, AA, BB, AFR participated in the design of the protocol and contributed to drafting the manuscript. All authors read and approved the final manuscript.

## Supplementary Material

Additional file 1 **Search strategy.** Search terms for MEDLINE and EMBASE databases. Click here for file

Additional file 2 **Data abstraction form.** Contains data abstraction tables, and risk of bias assessment table. Click here for file
