# Supplementary material for: Intra-aortic balloon pump in patients with cardiogenic shock complicating myocardial infarction: a systematic review and meta-analysis of randomized trials (protocol)
Source: Syst Rev. 2014 Mar 12;3:24. doi: 10.1186/2046-4053-3-24 (PMC4008254; doi:10.1186/2046-4053-3-24)
Supplement: Additional file 1 — Search strategy. Search terms for MEDLINE and EMBASE databases. [file 2046-4053-3-24-S1.docx]

Search Strategy:

1. exp cardiogenic shock/
2. exp shock/
3. exp heart left ventricle failure/
4. 2 and 3
5. 1 or 4
6. exp intraaortic balloon pump/
7. 5 and 6
8. exp clinical trial/ or clin$ trial$.mp.
9. exp Randomized controlled trial/
10. exp Randomization/
11. Single-Blind Method/
12. Double-Blind Method/
13. exp Random Allocation/
14. RCT.tw.
15. random$.mp.
16. (Single blind$ or Double blind$ or ((treble or triple) adj2 blind$)).tw.
17. comparative study/
18. controlled study/
19. Prospective study/
20. placebo:.mp.
21. 8 or 9 or 10 or 11 or 12 or 13 or 14 or 15 or 16 or 17 or 18 or 19 or 20
22. 7 and 21
