# Supplementary material for: Intra-aortic balloon pump in patients with cardiogenic shock complicating myocardial infarction: a systematic review and meta-analysis of randomized trials (protocol)
Source: Syst Rev. 2014 Mar 12;3:24. doi: 10.1186/2046-4053-3-24 (PMC4008254; doi:10.1186/2046-4053-3-24)
Supplement: Additional file 2 — Data abstraction form. Contains data abstraction tables, and risk of bias assessment table. [file 2046-4053-3-24-S2.docx]

IABP In Cardiogenic Shock Post Acute myocardial Infarction

| RefWork Number | | |  | | | |
| --- | --- | --- | --- | --- | --- | --- |
| Check Your Initials | | |  | | | |
| Full Journal Name | | |  | | | |
| Last Name of First Author | | |  | | | |
| Publication Year | | |  | | | |
| Study Design of RCT | | | □ Parallel  □ Other: -------------------- | | | |
| Was the study approved by an Ethics Committee or Institutional Review Board? | | | □Yes □ Not reported □ No | | | |
| Where did the study take place? (Check all that apply) | | | □ USA  □ Europe  □ Australia  Other : ----------------------- | | | |
| Was informed consent obtained from participants? | | | □ Yes □ Not reported □ No | | | |
| Funding Agency | | | □ Industry:..  □ other:.. | | | |
| Method of randomization | | | □ Random number table  □ Computer random-number generator  □ Coin tossing  □ Rolling of die  □ Picking allocation from a hat/box  □ Minimization/Dynamic allocation  □ Other description that contains elements convincing of appropriate generation of the allocation sequence (describe) .....................  □ Other description that contains elements convincing of inappropriate generation of the allocation sequence  (Describe)…………………………………..  □ No method described | | | |
| Check the box(s) of the clinical condition(s) being studied | | | □ mortality  □ Need for Inotrope and vasopressor  □ ICU length of stay (days)  □ Adverse events (stroke, limb ischemia,bleeding, Thrombocytopenia) | | | |
| Explicit diagnostic criteria used for determination of condition(s)? | | | □ Unequivocal clear and explicit criteria  □ Some criteria, but not as clear or explicit as desirable  □ Evident that explicit criteria were not used  □ Uncertain/not reported  □ Reported in a prior publication  □ Diagnostic criteria were referenced  □ Diagnostic criteria were not referenced | | | |
| Description of diagnostic criteria | | |  | | | |
| Concealment of allocation? | | | | □ Central randomization  □ Coded medication containers  □ Numbered, opaque, sealed envelopes  □ Envelopes, but at least one of opaque, sealed, and numbered not specified  □ Other (list) ……………………..  □ Clearly not concealed  □ Unclear whether rigorous concealment method used | |  |
| Blinding | Patients | | | □ Definitely yes  □ Probably  □ Probably not  □ Certainly not | |  |
|  | Health care providers | | | □ Definitely yes  □ Probably  □ Probably not  □ Certainly not | |  |
|  | Data collectors | | | □ Definitely yes  □ Probably  □ Probably not  □ Certainly not | |  |
|  | Assessors of outcomes | | | □ Definitely yes  □ Probably  □ Probably not  □ Certainly not | |  |
|  | Data analysis | | | □ Definitely yes  □ Probably  □ Probably not  □ Certainly not | |  |
| Length of treatment phase  (Ignore please) | | | | Intervention:……days  Control:…… days | |  |
| Type of intervention | | | | □ IABP  □ Medical therapy (definition): | |  |
| Number of individuals approached to take part in the study who choose not to participate | | □ Not reported  □ Reported as: ………………… | | | |  |
|  | | IABP  (n = ) | | | Control  (n = ) |  |
| Mean/Median age of patients | |  | | |  |  |
| Sex (M:F) | |  | | |  |  |
| APACHE II | |  | | |  |  |
| Anterior MI | |  | | |  |  |
| RV infarction | |  | | |  |  |
| Mechanical complication (VSD, acute MR) | |  | | |  |  |
| Received PCI | |  | | |  |  |
| Received Thrombolysis | |  | | |  |  |
| History of CAD | |  | | |  |  |
| Number not followed at all | |  | | |  |  |
| Number lost part-way through | |  | | |  |  |
| Number followed but not included in primary analysis | |  | | |  |  |
| Number followed but not included in secondary analysis | |  | | |  |  |
| How was lost to follow-up dealt with? | | □ Best/Worst case scenario  □ Counted as not having events, and included in the denominator  □ Exclusion from both the numerator and denominator  □ Last known value carries forward  □ Other: ……………………….. | | | |  |

| Follow-up | Time |
| --- | --- |
| Time | Days ……. |
| On treatment | □ Yes  □ No  □ uncertain |

Is there a potential conflict of interest?

□ Yes □ No □ uncertain □ Not reported

DEFINITIONS:

How were the following defined? And how was it captured (i.e. frequency and method for screening for the outcome)?

|  | Definitions | Methods of screening for outcomes |
| --- | --- | --- |
| Control Group  (Please describe in details how is control group defined) |  |  |
| Cardiogenic Shock  (Please describe how was cardiogenic shock defined in the study) |  |  |
| Mortality at 30 days |  |  |
| Mortality at 90 days |  |  |
| Hospital mortality |  |  |
| Vasopressors/inotrops(dose/duration) |  |  |
| Limb Ischemia |  |  |
| Clinically significant bleeding |  |  |

OUTCOMES:

Please indicate if outcome:

Not mentioned by letter ‘X’ and if not calculable by NC, Wright the exact number or if not available the % of patients with outcome between brackets with % sign following.

| Outcomes | N(%) | N(%) |
| --- | --- | --- |
| Mortality (at ) |  |  |
| ICU length of Stay (mean, SD) |  |  |
| Stroke (ischemic or hemorrhagic) |  |  |
| Thrombocytopenia. |  |  |
| Limb ischemia |  |  |
| Clinically significant bleeding: any bleeding that requires transfusion of more than two units of blood |  |  |

**Cochrane Risk of Bias tool**

| Domain | Description | Judgment |
| --- | --- | --- |
| Random sequence generation (selection bias) |  | Was allocation sequence adequately generated?  YES/NO/UNCLEAR |
| Allocation concealment (selection bias) |  | Was allocation concealed?  YES/NO/UNCLEAR |
| Blinding of participants and personnel (performance bias)  Mortality |  | Was blinding adequate?  YES/NO/UNCLEAR |
| Blinding of participants and personnel (performance bias) All outcomes |  | Was blinding adequate?  YES/NO/UNCLEAR |
| Blinding of outcome assessment (detection bias) Mortality |  | Was blinding adequate?  YES/NO/UNCLEAR |
| Blinding of outcome assessment (detection bias) Other outcomes |  | Was blinding adequate?  YES/NO/UNCLEAR |
| Incomplete outcome data (attrition bias) |  | Was incomplete outcome data addressed?  YES/NO/UNCLEAR |
| Selective reporting (reporting bias) |  | Were all pre-defined outcomes reported?  YES/NO/UNCLEAR |
| Other sources of Bias |  | No other biases?  YES/NO/UNCLEAR |

Further information required from author? Yes / No

Information requested

| 1 |  |
| --- | --- |
| 2 |  |
| 3 |  |
| 4 |  |
| 5 |  |
| 6 |  |

Corresponding author email address:

Date info requested: ... Date info received: ...
